# Iranian Patients Require More Pertinent Care to Prevent Type 2 Diabetes Complications

**DOI:** 10.1155/2014/409391

**Published:** 2014-07-22

**Authors:** Abdolreza Shaghaghi, Ali Ahmadi, Hossein Matlabi

**Affiliations:** Health Education & Promotion Department, Faculty of Health, Tabriz University of Medical Sciences, Tabriz 5166614711, Iran

## Abstract

*Background*. Accurate care of patients with type 2 diabetes may reduce risk of complications. This study was conducted to envisage current status of cares that are provided for a sample of Iranian patients with type 2 diabetes and highlight the domains that need to be focused on in the country's national type 2 diabetes care program. *Methods*. Behavioral risk factors and diabetes related complications were investigated among 234 randomly selected type 2 diabetic patients residing in the city of Khoy, Northwest of Iran. Data were collected by a semistructured questionnaire in face to face or telephone interview. Proportions and confidence intervals of the observed difference were calculated by the Confidence Interval Analysis (CIA) software version 2.2.0. *Results*. Diabetes complications were evident amongst 67.2% of the patients. Inappropriate dietary pattern, insufficient physical activity, and anxiety were reported by 26.5%, 74.8%, and 69.7% of the respondents. Quality of life was reported to be affected in 94.6% of the respondents but its burden was significantly greater in females (*P* < 0.001, 95% CI of the difference: −0.75 to −0.53). *Conclusions*. The findings reflect discrepancies in providing the required care for the studied Iranian patients with type 2 diabetes to prevent their disease's complications.

## 1. Background

Diabetes is one of the most prevalent chronic disorders in the world and more than 90% of the sufferers are affected with type 2 diabetes that is noninsulin dependent. The disease may provoke a number of serious and nonreversible complications in affected people [1]. Therefore, pathogenesis of the disease and its subsequent short and long term infirmities are amongst the world's most spotlighted health and medical issues [2]. Rapidly raising number of type 2 diabetes cases implies a global epidemic [3]. Worldwide prevalence of diabetes is estimated to be about 6.4%; hence, more than 280 million people are predicted to have diabetes in the world [4]. It is currently one of the fastest growing chronic complications in the world as annually 7 million new cases of diabetes are recognized and its complications have two victims in each ten seconds [5].

Prevalence of type 2 diabetes among 25–64-year-old Iranians was estimated to be 7.7% but only half of the cases have been diagnosed [3]. According to the reports of the International Diabetes Federation 70–80% of the Iranian type 2 diabetic patients are generally not aware of their illness in the early stages of the disease and after progress of the disease and confronting with its serious complications they become familiar with their disturbing disorder. Delayed diagnosis of the diabetes cases could lead to more serious and nonreversible complications and therefore induce enormous costs for both influenced patients and larger society [6]. Most frequent age range of type 2 diabetes diagnosis in Iran is 40–50, while expected age for onset of the disease in the developed world is often after the age of 65 [7]. This pattern is signaling diabetes commencement 15–25 years earlier than the age pattern existing for developed countries.

Rapidly growing number of type 2 diabetes in Iran which is consistent with the global epidemic could be attributable to the life styles changes including reduction of physical activity, change in diets, increase of obesity, and environmental stressors. According to the estimates of the World Health Organization (WHO) the number of type 2 diabetes in Iran will be more than 6 million by 2030 [5]. With current level of increase in life expectancy, occurrence of type 2 diabetes in early ages will lead to escalation of the disease related disability-adjusted life years (DALYs) in Iran.

The patients' quality of life, prevention of serious complications, and progress of the consequent disabilities completely depend on early diagnosis of the disease and proper management of the patients. Persuasion of the patients to adopt required changes in their lifestyle will have a pivotal role in diminishing the risks these patients may have in relation to the severely limiting or even life threatening complications of type 2 diabetes.

The issue of quality of provided health cares for diabetic patients was examined in different countries of the world [8–10]. These studies have concluded that a better diabetes care can be provided for the patients than hitherto. Importance of quality care for type 2 diabetes sufferers in Iran was also discussed in several studies [11–15].

While a written diabetes management and control program has been drafted in Iran and a number of diabetes clinics were established in major cities within the national health care system to provide health cares for patients suffering from type 2 diabetes, yet an updated research evidence do not exist to provide overall picture from the achievements these clinics had in terms of management of the disease, prevention of its complications, or making needed behavioral changes among the affected people.

This study was conducted to envisage current status of cares that are provided for a sample of Iranian patients with type 2 diabetes and also discuss pros and cons in providing the required quality care and highlight the behavioral domains that must be focused in the country's national diabetes care program.

## 2. Methods

### 2.1. Ethics Statement

This study has been approved by the Board of Ethics Committee of the Tabriz University of Medical Sciences (approval number: 800/4/5-16/04/2012).

### 2.2. Study Design and Participants

Stratified sampling method was used in this population based cross-sectional study to recruit 234 male and female type 2 diabetes patients from a list of 600 registered type 2 diabetes cases in the only diabetes clinic of the city of Khoy in North West of Iran which was a typical diabetes clinic within the country's Health Care System.

A semistructured questionnaire was used to collect data about the existent behavioral risk factors, diabetes related complication(s), and the participants' knowledge about actions that must be taken to avoid diabetes complications from August to October 2012. Content validity of the applied questionnaire consisting of 72 questions in seven domains of sociological and personal factors, risk factors before the disease diagnosis, the disease diagnosis and follow up, complications of the disease, current health status and knowledge about the potential risks, and the respondents point of views about the study questionnaire was assessed by a panel of experts including twelve endocrinologists and general practitioners. Their comments on the questionnaire were considered and reflected on the final draft. Reliability of this final draft was examined using Cronbach's alpha coefficient (*α* = 0.74).

### 2.3. Data Collection

The patients with active file in the clinic and those who were willing to give their informed consent to participate in the study were eligible to be recruited. Those patients who were not accessible after three telephone calls and those who were not able to or not interested to participate in the study were excluded.

The study questionnaire was filled through face to face interview within a private room or telephone interview for each patient where appropriate. To identify any pitfall in design of the questionnaire or data collection setting after completing the first 20 questionnaires in the data collection stage, we asked the participants to give their idea about the questionnaire and interview setting but nothing improper was pinpointed.

### 2.4. Data Analysis

Descriptive statistics were applied to analyze and report the study data. Proportions were used to summarize data for binary variables and confidence intervals for the observed difference in the proportions were calculated based on the recommended method of Newcombe and Altman [16] using the Confidence Interval Analysis (CIA) software version 2.2.0.

## 3. Results

### 3.1. Background Characteristics

The study participants' age, sex, level of education, place of residence, duration of having type 2 diabetes, and familial history of the disease were presented in Table 1.

Imbalance between the number of recruited male and female patients in the study sample is reflecting a major difference in the number of registered males and females having type 2 diabetes in the study center.

### 3.2. Health Care Received

Route of diabetes diagnosis and the health care that has been received by the studied patients were indicated in Table 2.

The observed difference between proportion of males and females in relation to the all five areas of received health care was statistically significant (*P* < 0.001). As presented, a larger number of female patients were reported to use glucometer regularly to check their blood glucose level but a relatively higher proportion of the males declared that they have received other proposed health care in this study.

### 3.3. Diabetes Complications

Presence of directly type 2 diabetes related complications, their type, and time of the diagnosis among the studied patients were shown in Table 3. Development of type 2 diabetes complications in about 32.8% of the studied patients before being diagnosed with the disease is worthy to be noticed but their occurrence in about 67.2% of the patients after being diagnosed may prove that the gaps already exist in providing quality cares to these patients.

Pattern of the onset of type 2 diabetes related complications except in relation to the proportion of male and female respondents who had the complications before their diabetes diagnosis and those who had neuropathic and retinopathic complications (*P* < 0.001) was significantly different in two sexes.

### 3.4. Behavioral and Nonbehavioral Risk Factors

Prevalence of the behavioral and nonbehavioral risk factors among the study participants that potentially could precipitate type 2 diabetes complications was indicated in Table 4.

The data represent the unmet needs at least in relation to the amendable risk factors of the disease complications. Excluding inappropriate dietary pattern the studied men and women indicated a significantly different behavioral and nonbehavioral risk factor profile of diabetes related complications (*P* < 0.001).

### 3.5. Knowledge about the Diabetes Complications

Knowledge of the studied patients about the important complications of the diabetes and their insight towards their quality of life was shown in Table 5.

The proportion of the respondents who had appropriate knowledge about the eye and cardiovascular complications and diabetic foot ulceration was significantly different in two sexes (*P* < 0.001) but no statistical differences was observed regarding the knowledge about renal complications of diabetes. Insight of the respondents in relation to the changes diabetes has created in their quality of life also was different between two sexes (*P* < 0.001, 95% CI of the difference: −0.75 to −0.53).

## 4. Discussion

### 4.1. Diabetes Care

Despite the emphasis that has been placed in the previous studies on the importance of self-care training in preventing diabetes complications [17] only a small number (6.4%) of the study participants reported to receive the mentioned trainings. The study findings indicated that a significantly higher proportion of men reported to receive the required cares after being diagnosed including self-care training and nutritional education compared to women. Such a discrepancy may reflect a real difference in receiving the diabetes related care between two sexes or only results from the different level of knowledge the study respondents had about the type and quality of health services they have to receive.

### 4.2. Heterogeneity of the Registered Patients with Type 2 Diabetes

The difference in the number of male (*n* = 168, 28.0%) and female (*n* = 432, 72.0%) registered patients in the only diabetes clinic of the study location (Khoy) also warrants further speculation. Findings of the study of Hagaieg et al. [18] and a recent meta-analysis on the prevalence of diabetes in Iran indicated that Iranian women are in higher risk of type 2 diabetes compared to men [19] but the observed difference in the number of registered men and women in the studied diabetes clinic which was the only diabetes clinic in the city was suggesting that the gap between two sexes should be much wider. However, probability of under representation of the male patients in the studied diabetes clinic could not be ruled out completely.

### 4.3. Diabetes Complications

Only about 10.7% of the study participants reported to be checked periodically for diabetes complications but the proportion of the male respondents who reported their routine assessment for the complications was significantly higher than the females. This may be due to the relatively higher proportion of the male diabetic patients who had cardiovascular complications and foot ulcers. The overall presence of diabetes related complications, however, was proportionately higher among females than males (*P* < 0.05). Such a difference in having two or more concurrent diabetes related complications between the sexes was also considerable.

### 4.4. Behavioral and Nonbehavioral Risk Factors

Suffering from disease related anxiety and having insufficient physical activity were highly prevalent among the study participants especially among females which is consistent with the findings of a previously conducted study [20]. This may results from a higher level of background insecurities females experience in Iran compared to men and limitations they have in doing physical activities in a traditionally male dominant society.

The studied men had a worse profile than the women regarding smoking and being obese. Doing housework including cooking and keeping house tidy and clean in the Iranian traditional society is the duty of housewives. This may have a positive protective effect on the women to prevent obesity especially in the older ages. In addition there is a social stigma against female smokers in Iran which explains a higher number of smoking males in the study compared to women.

### 4.5. Knowledge about Diabetes Complications

The study participants have revealed relatively a good level of knowledge about diabetes related health problems except for renal complications. This level of knowledge may result from experiencing the complications by the study participants as discussed above rather than the outcome of the educational programs planned for the patients. This conclusion is consistent with the scant number of the respondents who have reported their attendance in self-care training courses. An insufficient level of knowledge about the disease complications among the Iranian type 2 diabetes patients also was shown in the previous studies [21, 22]. Such an inconsistency is concurrent with the findings of an earlier study that reflected the inherent deficiency in the Iranian type 2 diabetes care protocol [23].

### 4.6. Quality of Life

The negative effect of the disease on the patients' quality of life was reported by 75.6% of the study participants of which most were women (88.7%). We have not checked in this study the perception of the study participants about quality of life and so the distinction between two sexes must be interpreted by caution. Delvarianzadeh et al. [24] have indicated in their study the impact of nutritional counseling in improving the perceived quality of life by the type 2 diabetic patients which may explain low perceived quality of life by the study participants where only 13.7% of them reported to receive educational counseling after being diagnosed with the disease.

### 4.7. The Study Limitations

Despite all efforts that were made in this study to comply with the internal and external validity criteria of a descriptive research probability of selection bias should not be ruled out. While the studied diabetes clinic was a typical center within the country's health system there is, however, possibility that the recruited patients in this study were not representative of all type 2 diabetes sufferers in the study location or Iran. However, the study findings could be considered as exploratory pilot study results that might guide a larger scale or multicenter research in future.

The high number of female registered patients in the studied diabetes clinic may reflect the limitations Iranian diabetic women had in access to the facilities of private health centers mainly because of economic burden. It is also important to perceive why, in spite of having high levels of type 2 diabetes complications, the studied women have reported receiving required cares in a lesser degree compared to men. This may be explained by limited request for the cares by the women due to the factors such as lack of time in view of their role in a traditional society as care giver at home or cultural barriers that limit their attendance at health care centers to receive their required cares. Further investigations are needed to fully understand the rationales behind the observed differences between males and females in relation to the diabetes complications, their knowledge about these complications, the health care they have received, and their insight towards effect of the disease on their quality of life.

Respondent bias as a result of the number of applied questions (72) for data collection and alternative data collection method (telephone interview) was another potential source of bias in this study since they might cause the study participants to be strenuous and so unable or unwilling to answer the study questions precisely.

## 5. Conclusions

The study findings indicated major discrepancies in providing the required care for the studied Iranian patients with type 2 diabetes to manage their disease and prevent the complications, while importance of empowerment of patients having type 2 diabetes in prevention of the disease complications was emphasized earlier in Iran [18]. Identified behavioral risk factors among the Iranian patients in this study revealed that the required empowerment was not created at least for the study participants and that missed puzzle pieces exist in the Iranian national type 2 diabetes care program. The investigation disclosed the importance of a prompt measure to intervene for early diagnosis of the disease explicitly in the study location and implicitly in the whole country.

## Figures and Tables

**Table 1:** Background characteristics of the studied type 2 diabetic patients.

Variables	Number	Percent
Age		
Less than 55 years old	131	56.0
Above 55 years old	103	44.0
Sex		
Male	68	29.1
Female	166	70.9
Place of residence		
Urban	92	39.3
Rural	142	60.7
Duration of having type 2 diabetes		
Less than 5 years	50	21.4
More than 5 years	184	78.6
Type 2 diabetes in the first degree relatives	86	36.8

**Table 2:** Route of diagnosis and received health care amongst the studied type 2 diabetic patients.

Variables	Number (%)		
	Male	Female	Total
Route of diabetes diagnosis			
Inactive: in a physician visit	63 (92.6)	158 (95.2)	221 (94.5)
Active: in the health care system screenings	5 (7.4)	8 (4.8)	13 (5.5)
Type of health cares received by the study participants			
Self-care training courses	11 (16.2)	4 (2.4)	15 (6.4)
Nutritional educations	21 (30.9)	11 (6.6)	32 (13.7)
Regular use of glucometer	26 (38.2)	104 (62.7)	130 (55.6)
Periodic checkups for complications	12 (17.6)	13 (7.8)	25 (10.7)
Current use of medications	45 (66.2)	71 (42.8)	116 (49.5)

**Table 3:** Patterns of the type 2 diabetes related complications in the studied patients.

Variables	Number (%)		
	Male	Female	Total
Presence of diabetes related complications	48 (70.6)	141 (84.9)	189 (80.8)
Presence of two or more concurrent diabetes complications	18 (26.5)	115 (69.3)	133 (70.37)
Time of the diabetes related complication(s) diagnosis			
Before being diagnosed with diabetes	19 (27.9)	43 (25.9)	62 (32.8)
After being diagnosed with diabetes	29 (42.6)	98 (59.0)	127 (67.2)
Type of diabetes related complication(s)			
Neuropathy	45 (66.2)	97 (58.4)	142 (75.0)
Retinopathy	21 (30.9)	57 (34.3)	78 (41.4)
Cardiovascular complications	30 (44.1)	10 (6.0)	40 (21.1)
Foot ulcers	7 (10.3)	3 (1.8)	10 (5.1)

**Table 4:** Prevalence of behavioral and nonbehavioral risk factors of type 2 diabetes related complications amongst the studied patients.

Variables	Number (%)		
	Male	Female	Total
Obesity	15 (22.1)	19 (11.4)	34 (14.5)
Smoking	36 (52.9)	12 (7.2)	48 (20.5)
Inappropriate dietary pattern	19 (27.9)	43 (25.9)	62 (26.5)
Insufficient physical activity	25 (36.8)	150 (90.4)	175 (74.8)
Anxiety	12 (17.6)	151 (91.0)	163 (69.7)

**Table 5:** Knowledge of the studied type 2 diabetic patients about complications of the disease and their insight towards their quality of life.

Variables	Number (%)		
	Male	Female	Total
Knowledge about:			
Eye complications	53 (77.9)	156 (94.0)	209 (89.3)
Foot ulcers	50 (73.5)	153 (92.2)	203 (86.8)
Cardiovascular complications	28 (41.2)	108 (65.1)	136 (58.1)
Renal complications	11 (16.2)	31 (18.7)	42 (17.9)
Insight towards			
Change in quality of life	20 (29.4)	157 (94.6)	177 (75.6)
